# Influence of Climate on Pulmonary Consumption

**Published:** 1843-06-10

**Authors:** 


### INFLUENCE OF CLIMATE ON PULMONARY CONSUMPTION.

M. Casimir Broussais read a short paper on this interesting subject. The author's conclusions are founded on the sanitary reports addressed by the surgeons attached to the forces in all parts of France and Africa to the military board of health. From the African reports it would appear, that of 50,341 patients who were carefully examined, only 62 labored under phthisis, giving a ratio of 1 to 650; and only 1 death from phthisis to 102 from all other diseases. Now, the ordinary mortality of the French army from phthisis is 1 to every 5 deaths from other diseases, as has been shown by the researches of M. Bensiston de Châteauneuf.

This difference is so great, and has been deduced from so large a number of cases, that if it should be shown to be constant, the beneficial effects of the African climate in cases of phthisis may be considered as proven. M. Broussais does not conceal the objections which may be offered to any conclusions drawn from his facts. The principal are contained in the following questions:—Are the African troops, which furnish a mortality from phthisis of 1 in 102, of the same class as those which in France give a mortality of 1 in 5? Are they not selected troops, stronger, &c.? Finally, is not the mortality from phthisis replaced by some other which cuts off those whom phthisis would have attacked at a subsequent period had they survived.

M. Broussais examines and discusses these objections in succession, and concludes,—

1. That no statistical documents prove the frequency of phthisis in India.

2. That the mortality from phthisis amongst the English soldiers in the West Indies is small, and one-fourth less amongst Europeans than black or colored people.

3. It has not been proved that pulmonary consumption is frequent in Martinique, Senegal, Cayenne, or Italy; on the contrary, the imperfect statistics which we possess seem to show the contrary.—*Trans. Acad. Med. of Paris, from Prov. Med. Journ.* April 15.

